# Determinants of minimum dietary diversity for lactating and pregnant women

**DOI:** 10.1371/journal.pone.0309213

**Published:** 2024-10-03

**Authors:** Md. Zakir Hossain, Md Jamal Uddin, Md Mizanur Rahman, Md Shahab Uddin, Toufique Ahmed

**Affiliations:** 1 Department of Statistics, Shahjalal University of Science & Technology, Sylhet, Bangladesh; 2 Team Leader of 5^th^ Year Annual Evaluation of JANO Project, Sylhet, Bangladesh; 3 Team Member (Data Analysis Expert) of 5^th^ Year Annual Evaluation of JANO Project, Sylhet, Bangladesh; 4 Faculty of Graduate Studies, Daffodil International University, Savar, Dhaka, Bangladesh; 5 Cooperative for Assistance and Relief Everywhere (CARE) Bangladesh, Dhaka, Bangladesh; St. Paul’s Hospital Millennium Medical College, ETHIOPIA

## Abstract

**Introduction:**

Maternal and child health, which is integral to public health, depends on maintaining a healthy diet during pregnancy and lactation to achieve optimal outcomes. This study aimed to investigate the prevalence and determinants of minimum dietary diversity (MDD) among pregnant and lactating women (PLW) in this particular context.

**Methods:**

A stratified cluster sampling approach was employed, encompassing intervention areas (Rangpur and Nilphamari in Bangladesh) as strata, with 30 clusters. The study included 631 pregnant and lactating women (PLW) aged 15 to 49 years, focusing on their consumption of a minimally diverse diet. The outcome variable was binary: MDD (1 = if they consumed ≥ 5 food items from a basket of 10 food groups, indicating they met the MDD; 0 = if they consumed < 5 items, indicating they did not meet the MDD), assessed based on ten food groups over a 24-hour period. The data were analyzed using a binary logistic regression model.

**Results:**

The study found that 51.19% of PLW met MDD criteria, indicating positive dietary practices. Those aged 21–49 years had significantly lower odds of meeting MDD than those aged 15–20 years. Education played a key role, with completion of primary (p = 0.029) and secondary incomplete education (p = 0.055) associated with higher odds of meeting MDD. Other identified predictors included climate-smart techniques for agriculture, women’s empowerment, food security, producing legume, nut and seeds and negative impact on family expenditure due to increase in commodity prices, especially food prices. Employing climate-smart agriculture increased odds by 1.58 times (p = 0.028), empowered women had 2.31 times higher odds (p < 0.001), and food security played a crucial role (p = 0.006). Moreover, producing legumes, nuts, or seeds was significantly associated with higher odds of meeting MDD (OR = 1.55, p = 0.039), while experiencing negative economic impacts lowered the odds (OR = 0.63, p = 0.034).

**Conclusion:**

The study provides insights into factors influencing MDD among PLW in northern Bangladesh. Empowering women and promoting climate-smart techniques for agriculture emerged as pivotal determinants, alongside enhancing education levels, increasing food security, and addressing economic barriers. Implementing multifaceted interventions that consider socio-demographic diversity, economic disparities, and dietary preferences is crucial for enhancing dietary diversity and improving health outcomes in this vulnerable population.

## Introduction

Maternal and child health is a crucial aspect of public health, and healthy eating during pregnancy and lactation is essential for the well-being of both women and their babies [1]. Sufficient dietary diversity during these critical times is necessary to prevent micronutrient deficiencies and inadequate nutrient intake, ensuring the best possible health outcomes for mothers and their children [2]. The lack of dietary diversity results in low energy and protein intake, further exacerbating the risk of malnutrition among women of reproductive age [3].

Despite notable advancements in a number of socioeconomic metrics, malnutrition remains a problem in Bangladesh, especially for PLW [4]. This scenario is more prevalent in the northern areas of Bangladesh [4, 5]. Previous studies in Bangladesh have shown that a considerable proportion of women of reproductive age do not meet the minimum dietary diversity (MDD) threshold, leading to micronutrient deficiencies [2, 4, 6, 7]. Moreover, according to the Bangladesh Demographic and Health Survey (BDHS) 2017–18, approximately 22% of women of reproductive age suffer from chronic energy deficiency, and an alarming 22% of newborns exhibit low birth weight, indicating a persisting public health challenge [8]. Therefore, malnutrition during pregnancy and lactation not only jeopardizes the immediate health of mothers but also has long-term implications for the growth and development of their children.

Northern Bangladesh, particularly Rangpur division, often faces unique challenges due to factors like poverty [9], limited access to healthcare [10], and environmental influences [11]. These challenges compound the difficulties faced by PLW in attaining a diverse and nutritious diet. Understanding the prevalence of MDD and its determinants in this specific region is crucial for tailoring effective interventions that address the unique needs of the population.

Several factors significantly influence dietary diversity among different demographic groups. For lactating mothers, factors such as formal education, decision-making autonomy, home gardening skills, nutrition knowledge, food security, and higher wealth indices play crucial roles in promoting better dietary diversity [3, 12]. These factors help to create a diverse and nutritious diet that benefits both maternal health and infant development. Conversely, a lack of maternal education, lower wealth status, and insufficient antenatal care visits are common risk factors associated with poor dietary diversity among children, which can also negatively impact maternal dietary practices [13, 14]. Addressing these factors holistically is essential for improving overall nutritional outcomes of this vulnerable populations [15].

This study aimed to investigate the prevalence and determinants of MDD among PLW in the northern part of Bangladesh, shedding light on the challenges and opportunities for improving maternal and child nutrition in this specific geographic context.

### Conceptual framework

**Fig 1:**
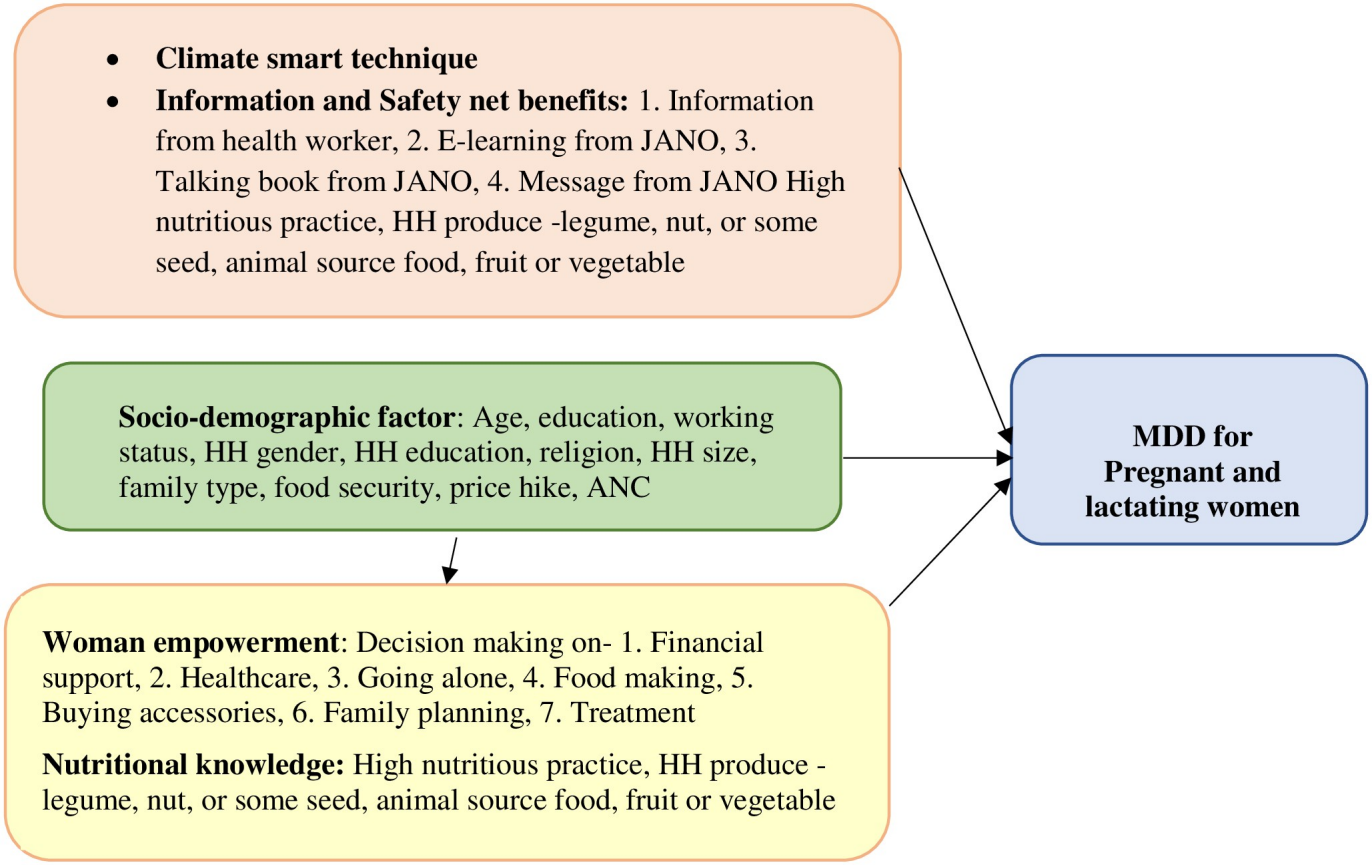
Minimum dietary diversity for women: A conceptual framework.

MDD-W (Minimum Dietary Diversity for Women) is a widely recognized indicator used to assess the adequacy of nutrient intake among pregnant and lactating women (Fig 1) [16]. It assesses whether women between the ages of 15 and 49 have ingested a minimum of five out of ten food groups within the past 24 hours [12, 17].

The concept, endorsed by organizations such as the World Health Organization (WHO) and the Food and Agriculture Organization (FAO), highlights the importance of consuming a variety of foods from different food groups. The MDD-W comprises ten food groups: grains, roots, and tubers; legumes and nuts; dairy products; meat, poultry, and fish; eggs; dark green leafy vegetables; other vitamin A-rich fruits and vegetables; other vegetables; other fruits; and fats and oils [18, 19].

Achieving MDD is critical for meeting the increased nutritional demands during pregnancy and lactation [20]. A diverse diet ensures a broad spectrum of essential nutrients, including vitamins and minerals, crucial for fetal development, maternal health, and successful breastfeeding. Inadequate dietary diversity has been linked to adverse maternal and child health outcomes, such as low birth weight, preterm birth, and impaired cognitive development in infants [7].

While there are many studies on maternal nutrition, it is crucial to focus specifically on the northern regions of Bangladesh due to their unique socio-economic, cultural, and environmental characteristics. Limited research has examined the dietary practices of PLW in these areas. By concentrating on the north, we aim to identify region-specific challenges and opportunities that can guide targeted interventions and policy recommendations.

Northern Bangladesh is predominantly agrarian, with a high prevalence of poverty and limited access to healthcare [9, 10]. These factors contribute to a complex web of challenges that impact dietary diversity among women. Additionally, cultural norms and dietary habits in this region may differ from the national average, necessitating a nuanced examination of the factors influencing dietary practices [21, 22].

Therefore, this paper seeks to contribute to the existing body of knowledge on maternal nutrition by providing insights into the determinants of MDD among pregnant and lactating women in northern Bangladesh. Through this investigation, we aim to inform evidence-based interventions and policies that address the unique challenges faced by women in this specific geographic context, ultimately enhancing maternal and child health outcomes.

## Methods

### Study setting (study area), design of the study, and study period

The study was implemented in two intervention districts in Bangladesh, Rangpur and Nilphamari, as part of the Joint Action for Nutrition Outcomes (JANO) project. JANO seeks to enhance the nutritional status of PLW, under five children, and adolescents; it primarily operates within government bodies and forums under the National Plan of Action for Nutrition 2 (NPAN-2). This cross-sectional study collected data through face-to-face interviews conducted from September to October 2023.

### Sampling procedures and sample size

The study utilized a stratified cluster sampling method, where Rangpur and Nilphamari districts were treated as separate strata, and villages within these districts served as clusters. Thirty clusters (intervention villages) were included in the household survey. These clusters were selected using a probability-proportional-to-size (PPS) systematic sampling approach, ensuring a representative sample weighted by total population size.

The study applied an established formula for determining sample size to estimate the number of households surveyed:

$$n=\frac{p(1-p)Z^{2}}{d^{2}}\times DE$$

Given a 46.9% indicator proportion (percentage of PLWs maintaining MDD), a Z-value of 1.96 (at 95% confidence level), a 0.05 margin of error, and assuming a design effect (DE) of 1.10, the sample size calculation indicates a need for at least 420 lactating women. For a comprehensive idea and in-depth analysis on the dietary diversity, the study included both lactating women and pregnant women. Therefore, the study has increased the sample size to 631 for covering a representative number of both lactating and pregnant women aged 15 to 49 years to assess the MDD. For distribution of these respondents in 30 clusters, 14 lactating women and 7 pregnant women were covered in each cluster.

### Data collection (methods and instruments)

For collection of necessary data and information on dietary diversity along with contextual factors, the study adopted a Household Survey. Fifteen enumerators, chosen and appointed by Data Management Aid, Dhaka, conducted the household survey. To ensure effective fieldwork, three supervisors and a field coordinator were hired. A five-day residential training session at BRAC Learning Centre, Rangpur, focused on technical aspects of the study and survey tools for data collection. The tools were developed prior to the training and finalized after piloting [23]. The data collection activities were closely monitored by the research team. The survey data was collected and stored electronically using SurveyCTO software [24].

### Inclusion and exclusion criteria of the respondents

The Household Survey involved participants comprising pregnant women at 3+ months of pregnancy and lactating mothers, aimed at examining the dietary habits of women of reproductive age in the designated districts. Households without any lactating mothers or without any pregnant women aged 15 to 49 years were excluded from the study (S1 Table, online supplements).

### Measurements (outcome measures, MDD)

A woman of reproductive age is classified as having consumed a minimally diverse diet if she ate at least five out of ten designated food groups during the last 24 hours (refer to S2 Table in the online supplements). The binary outcome variable, MDD, was assessed based on whether a mother consumed ≥ 5 food items from a specified basket of 10 food groups over a 24-hour period. A score of 1 was assigned if she met this criterion, indicating compliance with the MDD, while a score of 0 indicated consumption of < 5 items, signifying non-compliance of MDD. This scoring system enabled the evaluation of dietary diversity among participants within the study context. The study avoided duplicating counts within the same food group.

### Independent variables

The analysis considered important independent variables, as detailed in S3 Table in online supplements, which may be associated with MDD.

### Statistical analysis

At the initial phase of analysis, descriptive statistics were studied to gain insights into the characteristics of the variables. Categorical variables were explored through frequency distributions, and the percentage distribution of the outcome variable was depicted via pie charts, providing a visual representation of each category’s distribution within the dataset. To provide a more nuanced understanding, row percentages for each categorical variable were calculated, illuminating the proportional contribution of each category relative to the total observations. For continuous predictors, the mean and standard deviation (SD) were calculated, providing measures of central tendency and indicating the data’s dispersion, respectively. These descriptive statistics served as a foundational exploration of the dataset, offering a preliminary overview that laid the groundwork for subsequent analyses and model development.

The inferential approach used in this study included a two-step process to identify and select significant predictors for the multiple logistic regression model predicting the likelihood of meeting the MDD criteria. Initially, univariate logistic regression models were fitted for each independent variable individually, enabling examination of their individual associations with the outcome. A P-value cut-off of 0.25 was then applied to select variables for inclusion in the subsequent multiple logistic regression model [25]. This criterion helped narrow down the set of potential predictors by retaining those with a more substantial likelihood of influencing the outcome. Additionally, to ensure the robustness of the multiple logistic regression model, checks for multicollinearity were performed. Variables with a variance inflation factor (VIF) below 3 were considered free from multicollinearity, indicating that each retained variable provided unique information to the model without redundant overlap. This systematic approach ensured a rigorous selection process, aiming to enhance the model’s interpretability and predictive accuracy by considering both individual variable significance and the absence of multicollinearity among selected predictors.

We used Receiver Operating Characteristic (ROC) curve to evaluate the discriminatory ability of the fitted logistic regression model to distinguish between positive cases (MDD = 1, meets minimum dietary diversity) and negative cases (MDD = 0, does not meet minimum dietary diversity). Key aspects of the ROC curve include the True Positive Rate (Sensitivity), which is the proportion of actual positives correctly identified by the model, and the False Positive Rate (1—Specificity), which is the proportion of actual negatives incorrectly identified as positives. The Area Under the Curve (AUC) provides a single measure of overall model performance, with an AUC of 0.5 indicating no discriminative ability (equivalent to random guessing) and an AUC of 1.0 indicating perfect discrimination. Moreover, we also used the Hosmer-Lemeshow test [26] to assess the adequacy of the logistic regression model. This method evaluates the discrepancy between observed and expected frequencies across deciles of the model’s predicted probabilities.

### Ethical statement

The research adhered to strict ethical guidelines to ensure the protection of participants. Written informed consent was obtained from all participants, who had the independent choice to participate and provide their information. Participants were provided with a written consent form detailing the study’s purpose and their rights. Confidentiality of the participants’ data was maintained throughout the study. Additionally, the study obtained necessary ethical approvals from the Ethical Review Committee (PHFBD-ERC) of the Public Health Foundation, Bangladesh (Ref: PHFBD-ERC-FP13/2023).

## Results

### Exploratory data analysis

**Fig 2:**
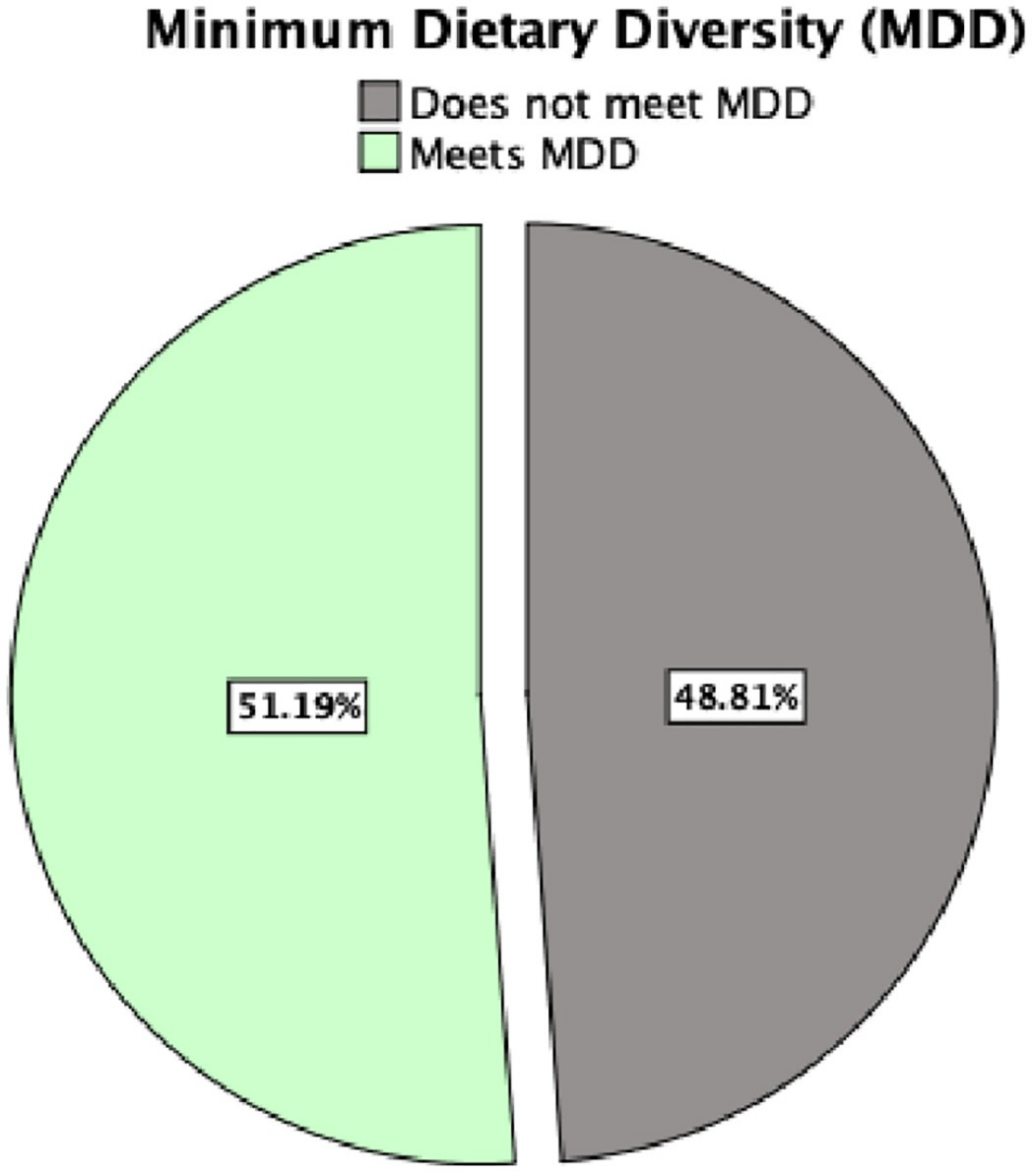
Distribution of respondents across minimum dietary diversity categories.

Fig 2 illustrates the distribution of respondents across the MDD categories and 51.19% of PLW met MDD criteria, signifying a bit higher dietary practice. Understanding these patterns is crucial for targeted interventions to enhance maternal and child nutrition.

Tables 1 and 2 show the frequency distribution of independent variables across the MDD categories. The sex of household heads revealed a trend, albeit not statistically significant (p = 0.07). Female-headed households demonstrated 73.3% not meeting the MDD, contrasting with 48.2% in male-headed households. Similarly, Hindu-headed households exhibited a higher percentage meeting the MDD (61.4%) than Muslim-headed households, though it was not statistically significant (p = 0.07). Age categories of PLW did not exhibit a significant association with meeting the MDD criteria. However, individuals aged 15–20 years displayed a higher percentage meeting the criteria (58.8%). Regarding the age of the household head, those aged >50 years demonstrated a higher percentage meeting the criteria (58.6%, p = 0.20). While education levels for PLW and household heads did not significantly influence meeting the MDD criteria, there was a discernible trend. Occupation, for both PLW and household heads, as well as the working status of PLW, did not show significant associations with meeting the MDD criteria (p = 0.87, p = 0.48, p = 0.56, respectively). Notable differences emerged based on the type of household (p = 0.001). Combined/Extended households had a higher percentage meeting the criteria (58.4%). Additionally, the number of rooms in the house significantly associated with meeting the criteria (p = 0.001), where households with three or more rooms had a higher percentage meeting the criteria (62.2%). Women empowerment emerged as a significant predictor, with empowered women associated with a higher percentage meeting the MDD criterion (p < 0.001).

**Table 1:** Frequency distribution of sociodemographic variables across the Minimum Dietary Diversity (MDD) categories (n = 631).

Variables	Categories	Minimum Dietary Diversity				
		Does not meet MDD		Meets MDD		p-value
		Count	Row (%)	Count	Row (%)	
Sex of HH Head	Male	297	48.2	319	51.8	0.07
	Female	11	73.3	4	26.7	
Religion of HH Head	Islam	281	50.1	280	49.9	0.07
	Hindu	27	38.6	43	61.4	
Age of PLW	15–20 year	70	41.2	100	58.8	0.11
	21–25 year	115	53.5	100	46.5	
	26–30 year	74	51	71	49	
	31–49 year	49	48.5	52	51.5	
Age of HH head	< = 30 year	112	51.1	107	48.9	0.20
	31–50 year	148	50	148	50	
	>50 years	48	41.4	68	58.6	
Education of PLW	Primary incomplete	37	51.4	35	48.6	0.60
	Primary complete	48	45.7	57	54.3	
	Secondary incomplete	131	50.6	128	49.4	
	SSC or HSC	70	44.9	86	55.1	
	Higher-Bachelor or Master	22	56.4	17	43.6	
Education of HH head	Primary incomplete	135	54.9	111	45.1	0.08
	Primary complete	56	43.4	73	56.6	
	Secondary incomplete	59	43.7	76	56.3	
	Secondary complete or above	58	47.9	63	52.1	
Occupation of PLW	House wife	258	49	269	51	0.87
	Other	50	48.1	54	51.9	
Occupation of HH Head	Farmer or Share Cropper	76	45.2	92	54.8	0.48
	Labour or worker	125	52.5	113	47.5	
	Service or business or self-employee or doctor	65	46.4	75	53.6	
	Others	42	49.4	43	50.6	
PLW Currently working Status	Yes	21	53.8	18	46.2	0.56
	No	256	48.9	267	51.1	
Type of household	Unit	187	55	153	45	0.001
	Combined/Extended	121	41.6	170	58.4	
HH size categories	2–3 person	63	51.6	59	48.4	0.75
	4–6 person	211	48.4	225	51.6	
	>6 person	34	46.6	39	53.4	
Rooms are there in the house	One Room	120	57.7	88	42.3	0.001
	Two Rooms	112	50.5	110	49.5	
	Three or more rooms	76	37.8	125	62.2	
Women Empowerment	No	255	52.8	228	47.2	<0.001
	Yes	53	35.8	95	64.2	
Number of ANC visit	1–3 visits	138	50.2	137	49.8	0.77
	4 visits	109	47	123	53	
	4+ visits	47	48.5	50	51.5	

**Table 2:** Frequency distribution of health and dietary related variables across the Minimum Dietary Diversity (MDD) categories (n = 631).

Variables	Categories	Minimum Dietary Diversity				
		Does not meet MDD		Meets MDD		p-value
		Count	Row (%)	Count	Row (%)	
HH practiced high nutrient product	No	143	58.8	100	41.2	<0.001
	Yes	165	42.5	223	57.5	
Climate smart technique	No	233	56.4	180	43.6	<0.001
	Yes	75	34.4	143	65.6	
Received any information on health and nutrition using Talking Book device of the JANO project	Yes	169	46.8	192	53.2	0.25
	No	139	51.5	131	48.5	
Received any e-learning apps from JANO project	Yes	53	39.8	80	60.2	0.02
	No	255	51.2	243	48.8	
Do the health workers conduct session through e-session apps using mobile phones/ laptop/tab?	Yes	117	45.9	138	54.1	0.65
	No	88	48.1	95	51.9	
Nutrition knowledge	No	125	53	111	47	0.11
	Yes	183	46.3	212	53.7	
Food Security	Secure Food	112	38.9	176	61.1	<0.001
	Normal	58	43.9	74	56.1	
	Moderate to severe	138	65.4	73	34.6	
Negative impact on your family expenditure due to the recent increase in commodity prices, especially food prices?	Yes	244	54	208	46	<0.001
	No	64	35.8	115	64.2	
Produce Zink rice	No	301	49.1	312	50.9	0.40
	Yes	7	38.9	11	61.1	
Produce legume, nut, and seeds	No	183	59.8	123	40.2	<0.001
	Yes	125	38.5	200	61.5	
Produce fruits and vegetable	No	149	61.8	92	38.2	<0.001
	Yes	159	40.8	231	59.2	
Produce color vegetable	No	200	58	145	42	<0.001
	Yes	108	37.8	178	62.2	
Food from animal source	No	193	49.9	194	50.1	0.50
	Yes	115	47.1	129	52.9	
Total homestead land for vegetable production	Mean (SD)	0.77 (1.77)		1.11 (1.90)		0.021

Several health and nutrition factors demonstrated varying associations with meeting the MDD criteria. ANC visits and information from the JANO project did not show significant associations (p = 0.77 and p = 0.25) whereas high nutrient product practice has significant association (p < 0.001) with MDD. However, the adoption of climate-smart agriculture techniques significantly influenced meeting the criteria (p < 0.001), as did receiving e-learning apps from the JANO project (p = 0.02).

Nutrition knowledge showed a trend but was not significantly associated with meeting the MDD criteria (p = 0.11). In contrast, food security significantly influenced meeting the MDD criteria (p < 0.001), with households facing moderate to severe food insecurity having a lower percentage meeting the MDD criterion. Negative economic impacts due to rising commodity prices significantly lowered the percentage meeting the MDD criteria (p < 0.001). Producing legumes, nuts, and seeds (p < 0.001), fruits and vegetables (p < 0.001), and colored vegetables (p < 0.001) were all significantly associated with meeting the MDD. Specifically, individuals who produced these food groups were more likely to meet the dietary diversity standards compared to those who did not. Additionally, the mean homestead land for vegetable production was significantly larger (p = 0.021) for those meeting the MDD, indicating that greater land allocation for vegetable production is positively correlated with better dietary diversity. These results underscore the importance of diverse food consumption and adequate land resources for vegetable production in achieving minimum dietary diversity.

### Inferential analysis (binary logistic regression)

Table 3 shows the parameter estimates, odds ratios, and 95% confidence intervals from the binary logistic regression model. The analysis aimed to investigate the determinants of meeting the MDD criteria (MDD = 1) within the studied population. Various variables were examined for their association with MDD, emphasizing those exhibiting statistical significance (P-value ≤ 0.05). Notably, odds ratios (OR) were considered alongside their corresponding 95% confidence intervals (CI) to provide a comprehensive understanding of the results.

**Table 3:** Parameter estimates, odds ratios (OR), and 95% confidence intervals (CI) for OR from the binary logistic regression model (outcome variable: Minimum Dietary Diversity (MDD criteria**).

Variables	Categories	Estimate	S.E.	P-value	OR	95% C.I. for OR	
						Lower	Upper
Sex of HH Head	Male*						
	Female	-1.160	0.637	0.068	0.313	0.090	1.091
Religion of HH Head	Hindu	0.179	0.289	0.537	1.195	0.678	2.107
	Islam*						
Age of PLW	15–20 year*						
	21–25 year	-0.529	0.232	**0.023**	0.589	0.374	0.929
	26–30 year	-0.307	0.255	0.230	0.736	0.446	1.214
	31–49 year	-0.336	0.283	0.235	0.714	0.410	1.244
Education of HH head	Primary incomplete (below primary)*						
	Primary complete	0.528	0.242	**0.029**	1.696	1.055	2.726
	Secondary incomplete	0.471	0.245	**0.055**	1.601	0.990	2.588
	Secondary complete or above	0.031	0.249	0.903	1.031	0.633	1.680
Type of household	Unit*						
	Combined/Extended	0.255	0.230	0.269	1.290	0.821	2.027
Climate smart-agriculture	No*						
	Yes	0.457	0.207	**0.028**	1.579	1.052	2.372
Women Empowerment	No*						
	Yes	0.835	0.224	**<0.001**	2.305	1.485	3.578
Nutrition knowledge	No*						
	Yes	0.093	0.189	0.621	1.098	0.759	1.589
Food Security	Secure Food*						
	Normal	0.068	0.245	0.781	1.071	0.663	1.730
	Moderate to severe	-0.626	0.226	**0.006**	0.535	0.343	0.833
Received any information on health and nutrition using Talking Book device of the JANO project	No	-0.064	0.182	0.726	0.938	0.656	1.341
	Yes*						
Rooms in the house	One Room*						
	Two Rooms	-0.195	0.246	0.429	0.823	0.508	1.334
	Three or more rooms	0.197	0.291	0.498	1.218	0.688	2.155
Produce legume, nut and seeds	Yes	0.436	0.211	**0.039**	1.546	1.023	2.336
	No*						
Produce fruits and vegetable	Yes	-0.054	0.242	0.823	0.947	0.590	1.522
	No*						
Produce color vegetable	Yes	0.421	0.218	0.054	1.524	0.993	2.338
	No*						
Negative impact on family expenditure due to increase in commodity prices, especially food prices	Yes	-0.464	0.218	**0.034**	0.629	0.410	0.965
	No*						
Area of total homestead land for vegetable production		0.011	0.051	0.829	1.011	0.915	1.118

*Reference category,

**MDD = 0 (Does not meet minimum dietary diversity) and MDD = 1(Meets minimum dietary diversity) HH: Household, PLW: Pregnant and Lactating Women; JANO: Joint Action for Nutrition Outcome.

In terms of household gender dynamics, female-headed households showed a trend towards 0.313 times lower odds of meeting MDD compared to male-headed households, although this trend was not statistically significant (P = 0.068). Religion of the household head and nutrition knowledge did not demonstrate significant associations with MDD. Regarding the age categories of PLW, individuals aged 21–49 years exhibited lower odds of meeting MDD compared to those aged 15–20 years. However, only the 21–25 years age group showed a significant impact on MDD (p = 0.023). The education level of the household head played a role, with completion of primary education significantly associated (p = 0.029) with higher odds of meeting MDD compared to the household head whose education was below primary (OR = 1.696, 95% CI [1.055, 2.726]).

Key predictors for MDD included climate-smart techniques, women’s empowerment, and food security. Specifically, households employing climate-smart techniques had 1.579 times higher odds of meeting MDD (P = 0.028), and empowered women were associated with 2.305 times higher odds of meeting MDD (P <0.001). Food security also played a crucial role, with households facing moderate to severe food insecurity having 0.535 times lower odds of meeting MDD (P = 0.006).

Concerning specific dietary choices, cultivating legumes, nuts, or seeds was significantly associated (p = 0.039) with higher odds of meeting MDD (OR = 1.546, 95% CI [1.023, 2.336]), while area of total homestead land for vegetable production; fruits, vegetables; and colorful vegetables did not exhibit significant associations. Additionally, experiencing negative economic impacts due to rising commodity prices significantly (P = 0.034) lowered the likelihoods of meeting MDD (OR = 0.629, 95% CI [0.410, 0.965]).

These comprehensive findings illuminate various socio-demographic, economic, and dietary factors influencing MDD, offering valuable insights for targeted interventions and policy strategies aimed at improving dietary diversity within similar demographic contexts.

The Receiver Operating Characteristic (ROC) analysis was conducted on the final logistic regression model with Area Under the Curve (AUC) (S1 Fig). The AUC, ranging from 0 to 1, quantifies the model’s ability to distinguish between individuals meeting and not meeting the MDD criteria. The ROC curve for the logistic regression model assessing the prediction performance for the MDD shows the AUC of 0.74. This indicates that the model has a good ability to distinguish between individuals with and without MDD. The curve demonstrates the trade-off between sensitivity (true positive rate) and 1-specificity (false positive rate) across different thresholds, with the model performing significantly better than random guessing, represented by the diagonal red line. Here the AUC of 0.74 falls within the range of acceptable discrimination ability, suggesting that the model is reasonably effective at predicting MDD.

Moreover, based on the Hosmer-Lemeshow test, the logistic regression model fits the data adequately, as the Chi-square value is 10.035 with 8 degrees of freedom (p-value = 0.263), which is greater than the conventional threshold of 0.05. This suggests that the model’s predicted probabilities align reasonably well with the observed frequencies of the outcome variable across different groups or bins of predicted probabilities.

## Discussion

Maternal health, particularly during pregnancy and lactation, is a vital public health concern as it directly affects newborn health. Ensuring adequate dietary diversity during these critical periods is essential for optimal health outcomes. This study aimed to uncover the prevalence and determinants of MDD, shedding light on challenges and opportunities for improving maternal and child nutrition in the northern regions of Bangladesh.

We found 51.19% of PLW met MDD criteria. The study revealed intriguing trends in gender dynamics within households, indicating that female-headed households exhibited a lower percentage of meeting the MDD compared to male-headed households [27] and while this trend was not statistically significant. In Bangladesh, female-headed households have higher dependency ratio than male-headed households, and the economic condition of female-headed households is poor than male-headed households [28]. Additionally, the religion of the household head showed a notable trend, albeit not statistically significant, suggesting the importance of exploring the influence of religion on dietary patterns within households in Bangladesh [29]. These findings underscore the complexity of gender dynamics and religious factors in shaping dietary outcomes within households, highlighting the need for more in-depth research to understand the nuances of these relationships and their implications for nutrition and food security in the country.

Age categories (except 21–25 years) of PLW did not show a significant association with the MDD criteria. Individuals aged 21–25 years displaying a significant lower odd of meeting the MDD criteria than the 15–20 years [17, 30]. Similar finding was observed a study conducted in Bangladesh by Shaun et al [17]. Additionally, the age of the household head, particularly those aged >50 years, demonstrated a higher percentage meeting the criteria but the association was not statistically significant [30]. Although education levels for PLW and household heads did not significantly influence meeting the MDD criteria in the bivariate analyses, in the multivariable logistic regression, there was a significant impact of the household head’s education (i.e., primary complete compared to primary incomplete) on meeting the MDD criteria. Previous studies highlight that the literacy of the household head is a major determinant in achieving high dietary diversity, which in turn impacts household food security [5, 31].

Occupation for PLW and household heads, as well as the working status of PLW, did not show significant associations with meeting the MDD criteria. However, the type of household and the number of rooms in the house may appear to be significantly associated with meeting the MDD criteria only in univariate analysis [32]. Still, in multivariable analysis, other variables may better explain the variance in MDD, reducing the apparent effect of the type of household [33] or the number of rooms in the house.

Empowered women emerged as a significant predictor, with higher odds of meeting the MDD criterion, as shown by other studies [34, 35]. Moreover, previous studies have highlighted that women’s decision-making autonomy, education level, and socioeconomic status play crucial roles in achieving higher dietary diversity scores [3]. Additionally, women’s empowerment, particularly in terms of social independence, has been linked to increased odds of meeting the MDD threshold, emphasizing the importance of empowerment in nutritional outcomes [36].

In terms of health and nutrition factors, ANC visits and information from the JANO project did not show any significant association with meeting the MDD. However, the adoption of climate-smart techniques for agricultural production significantly positively influenced meeting the criteria [37]. Previous studies showed that climate-smart techniques for agricultural production, such as cultivating flood and drought-resistant crops, using organic fertilizers, and implementing agroforestry, enhance productivity and resilience while reducing greenhouse gas emissions [38, 39]. Additionally, agricultural diversification positively impacts dietary diversity among farm households, highlighting the need to promote diversified agricultural production alongside women’s education, employment, and access to information [40]. Nutrition knowledge showed a trend but was not significantly associated with meeting the MDD criteria. Conversely, food security significantly influenced meeting the criteria, with households facing moderate to severe food insecurity having a lower percentage meeting the criteria, as affirmed by other studies [12, 41]. Negative impact on family expenditure due to increase in commodity prices, especially food prices, significantly lowered the percentage meeting the MDD criteria. Several studies, particularly in low and middle-income countries, highlight the correlation between economic constraints and the reduced ability of families to afford a variety of nutritious foods. This situation often leads to poorer dietary choices and can have significant health implications [42].

Several studies suggest that various dietary choices significantly influence meeting the MDD criteria, with notable associations observed for cultivating legumes, nuts, seeds, and colorful vegetables [43, 44]. As indicated in other studies, the mean homestead land for vegetable production significantly differed between those meeting and not meeting the criteria [12, 45]. However, in our study, even with only univariate analysis, this variable showed similar results to the previous studies.

### Strengths and limitations

One strength of this study is its comprehensive exploration of diverse factors influencing dietary diversity, covering a wide range of socioeconomic, demographic, and community-level determinants. The large sample size adds to the statistical power and reliability of the results, while the detailed analyses provide in-depth insights into the complexities of dietary diversity. These elements collectively enhance the robustness and generalizability of the findings.

However, the cross-sectional nature of the study inherently limits the ability to establish causality, as it captures data at a single point in time rather than over a prolonged period. This design restricts the capacity to infer temporal relationships between variables and MDD. Additionally, the reliance on self-reported data introduces the potential for recall bias, where participants may not accurately remember or report their dietary intake. This can lead to measurement inaccuracies that may affect the study’s conclusions.

### Recommendations

Based on the study results, several comprehensive recommendations can be made for policy makers to improve minimum dietary diversity among vulnerable populations. Interventions should be tailored to address the unique nutritional needs of pregnant and lactating women, with practical solutions such as community-based nutritional counseling and support programs that include providing supplements and educational awareness workshops on balanced diets and breastfeeding practices. Enhancing education levels among household heads, particularly ensuring the completion of primary education, can be achieved through adult education programs that integrate nutritional education with basic literacy and numeracy, incentivized by free meals or childcare.

Strengthening women’s empowerment initiatives is crucial, and this can be practically supported by establishing self-help groups that focus on income-generating activities, nutrition education, and community leadership training, along with access to microcredit facilities.

Encouraging the adoption of climate-smart agricultural techniques is essential for improving food security, which can be supported by providing training and resources for practices like conservation agriculture, offering subsidies, and financial incentives to smallholder farmers. Addressing food security challenges involves implementing food assistance programs that provide direct support to the most vulnerable populations, such as food vouchers, cash transfers, and food banks, alongside strengthening social safety nets. Promoting the production of diverse foods is vital, and can be facilitated by providing farmers with high-quality seeds and inputs for cultivating diverse crops, organizing training sessions on sustainable farming practices, and creating local markets and cooperatives to help farmers sell their produce.

Finally, mitigating the economic impacts of rising commodity and food prices is critical. This can be achieved by introducing price stabilization funds and food price monitoring systems, implementing targeted subsidies for essential food items, and providing financial support to low-income families through direct cash transfers or food stamps. By focusing on these interconnected areas and implementing these practical solutions, policy makers can develop comprehensive strategies that enhance dietary diversity and improve nutritional outcomes for vulnerable populations.

## Conclusion

In conclusion, this study provides a nuanced understanding of the factors associated with meeting MDD criteria among pregnant and lactating women in the northern part of Bangladesh. While some trends were not statistically significant, they offer valuable insights for tailored interventions and policy strategies. Addressing the identified determinants, particularly empowering women, promoting climate-smart techniques for agriculture, enhancing education levels, and implementing food security programs, holds promise for improving maternal and child nutrition in similar demographic contexts. Additionally, targeting specific age groups, promoting the production of diverse foods, and mitigating the economic impacts of rising commodity prices are crucial for enhancing dietary diversity. The take-home message emphasizes the importance of multifaceted interventions that consider socio-demographic, economic, and dietary factors to improve dietary diversity and, consequently, the health outcomes of pregnant and lactating women. These comprehensive strategies can significantly contribute to better nutrition and overall well-being in vulnerable populations.

## Supporting information

S1 Fig Receiver Operating Characteristic (ROC) curve for logistic regression model: Assessing MDD prediction performance (AUC = 0.74). (DOCX)

S1 Table Detailed criteria for inclusion of the respondents. (DOCX)

S2 Table Food groups for calculating minimum dietary diversity (MDD). (DOCX)

S3 Table Necessary explanatory variables associated with minimum dietary diversity (MDD). (DOCX)
