# Supplementary material for: Determinants of minimum dietary diversity for lactating and pregnant women
Source: PLoS One. 2024 Oct 3;19(10):e0309213. doi: 10.1371/journal.pone.0309213 (PMC11449314; doi:10.1371/journal.pone.0309213)
Supplement: S1 Fig — (DOCX) [file pone.0309213.s001.docx]

**Online supplements**


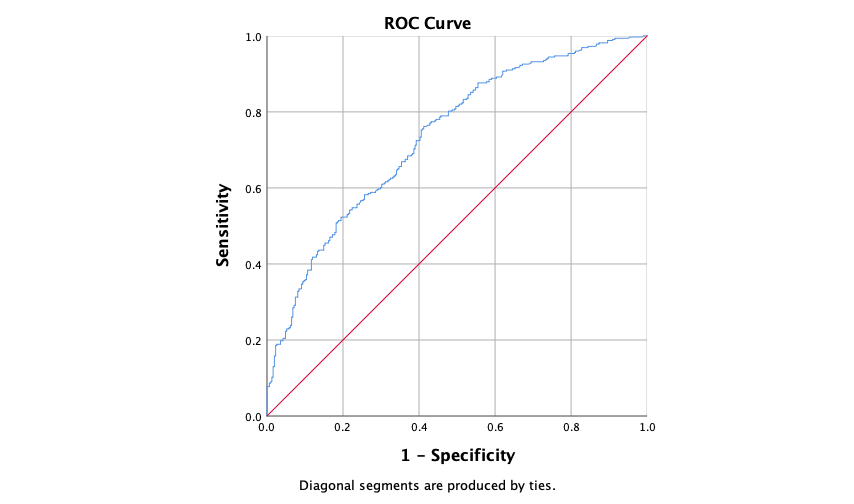


**S1 Figure:** Receiver Operating Characteristic (ROC) Curve for Logistic Regression Model: Assessing MDD Prediction Performance (AUC=0.74)
