# Supplementary material for: Determinants of minimum dietary diversity for lactating and pregnant women
Source: PLoS One. 2024 Oct 3;19(10):e0309213. doi: 10.1371/journal.pone.0309213 (PMC11449314; doi:10.1371/journal.pone.0309213)
Supplement: S1 Table — (DOCX) [file pone.0309213.s002.docx]

**Online supplements**

**S1 Table:** Detailed criteria for inclusion of the respondents

| Inclusion criteria | Respondents/ Participants |
| --- | --- |
| Women of reproductive age in the targeted districts who are consuming a minimum dietary diversity (MDD) | Pregnant and Lactating women aged 15 to 49 years |
| Participation of women, in formal (government led) and/or informal (civil society led, private sector led) decision making spaces | Pregnant and lactating women |
| Women have claimed nutrition-specific and sensitive services from relevant service providers | Pregnant and lactating women, Adolescent girls |
| PLW from the target population received nutrition specific safety net support | Pregnant and lactating women aged 15 to 45 years |
| Households involved in the production of higher-value nutrition products | Household head (Producers or farmers) |
