# Supplementary material for: Determinants of minimum dietary diversity for lactating and pregnant women
Source: PLoS One. 2024 Oct 3;19(10):e0309213. doi: 10.1371/journal.pone.0309213 (PMC11449314; doi:10.1371/journal.pone.0309213)
Supplement: S2 Table — (DOCX) [file pone.0309213.s003.docx]

**Online supplements**

**S2 Table:** Food groups for calculating minimum dietary diversity (MDD)

| Food groups | Items |
| --- | --- |
| Grains/roots/tubers | Grains (Rice, Wheat, Maize, Kawin), Roots and Tubers (bread, rice) |
| Pulse | Beans, peas, lentils |
| Dairy products | Milk, Yoghurt, Cheese |
| Meat poultry and fish | Meat and Fish (including dry fish) |
| Dark green leafy vegetables | Spinach, green chili, ladies’ finger, pumpkin leaves, sweet potato leaves, spinach |
| Vitamin-A rich fruits and vegetables | Ripen mango, ripen papaya, carrot, pumpkin, sweet potatoes, green mango, green papaya and vegetables |
| Others vegetables | Any type of vegetables |
| Other fruits | Any type of fruits |
| Nuts and seeds | Any type of nuts and seeds |
| Eggs | - |
