# Supplementary material for: Determinants of minimum dietary diversity for lactating and pregnant women
Source: PLoS One. 2024 Oct 3;19(10):e0309213. doi: 10.1371/journal.pone.0309213 (PMC11449314; doi:10.1371/journal.pone.0309213)
Supplement: S3 Table — (DOCX) [file pone.0309213.s004.docx]

**Online supplements**

**S3 Table:** Necessary explanatory variables associated with minimum dietary diversity (MDD)

| **Characteristic** | **Description** | **Type** |
| --- | --- | --- |
| Age of the PLW | Age of pregnant and lactating woman in years (PLW) | *Categorical*  15-20 year  21-25 year  26-30 year  31-49 year |
| Education of PLW | Education status of PLW | *Categorical*  Primary incomplete  Primary complete  Secondary incomplete  SSC or HSC  Higher-Bachelor or Master |
| Working status of PLW | Currently working status of PLW | *Binary*  Yes  No |
| Occupation of PLW | Types of occupation of PLW | *Categorical*  House wife  Other |
| Age of the Household head (HH) | Age of the Household head (HH) in years | *Categorical*  <=30 year  31-50 year  >50 years |
| Gender of HH | Gender of household head | Male  Female |
| Education of HH | Level of education of household head | *Categorical*  Primary incomplete  Primary complete  Secondary incomplete  SSC or HSC/Higher |
| Religion of HH | Religion of PLW | *Categorical*  Hindu  Islam |
| Family members (HH size) | Number of family members in a household | *Categorical*  2-3 person  4-6 person  >6 person |
| Family type | Types of family (join vs nuclear) | *Categorical*  Unit  Combined/Extended |
| Homestead gardening | Total amount of cultivated homestead land | *Continuous* |
| ANC | Number of ANC of PLW | *Categorical*  1-3 visits  4 visits  4+ visits |
| Food Security | Food security status of PLW | *Categorical*  Secure Food  Normal  Moderate to severe |
| Climate smart technique | Score of households practicing climate smart agricultural techniques | *Binary*  Yes  No |
| Empowerment | Maternal decision autonomy | *Binary*  Yes  No |
| High nutritious value | Households produce five categories of crops (bio-fortified, legume, nut, or some seed, animal source food including dairy products, dark yellow or orange-fleshed root or tuber, fruit or vegetable) | *Binary*  Yes  No |
| High nutrition practice | HH practice high nutrition | *Binary*  Yes  No |
| Nutritional Knowledge | Score of nutritional knowledge | *Binary*  Yes  No |
| Price hike | Negative impact of price hike on MDD | *Binary*  Yes  No |
| E-learning | E-learning from JANO | *Binary*  Yes  No |
| Access to information | Information receives from JANO | *Binary*  Yes  No |
